# MOG antibody seropositivity in a patient with encephalitis: beyond the classical syndrome

**DOI:** 10.1186/s12883-017-0971-6

**Published:** 2017-10-05

**Authors:** Sara Mariotto, Salvatore Monaco, Patrick Peschl, Ilaria Coledan, Romualdo Mazzi, Romana Höftberger, Markus Reindl, Sergio Ferrari

**Affiliations:** 1 Department of Neuroscience, Biomedicine and Movement Sciences, Section of Neurology, University of Verona, Verona, Italy; 2 Clinical Department of Neurology, Medical University of Innsbruck, Innsbruck, Austria; 3 Department of Diagnostics and Public Health, Section of Infectious Diseases, University of Verona, Verona, Italy; 4 Institute of Neurology, Medical University of Vienna, Vienna, Austria

**Keywords:** Autoimmune diseases, Encephalitis, Anti-myelin oligodendrocyte glycoprotein antibodies (MOG-abs)

## Abstract

**Background:**

The presence of circulating anti-myelin oligodendrocyte glycoprotein antibodies (MOG-Abs) has been described in sera of patients with different inflammatory conditions of the central nervous system. In adults the core clinical feature is usually characterised by acute myelitis and/or optic neuritis. We here report an atypical case with serum and cerebrospinal fluid MOG-Abs and a clinical picture suggestive for acute encephalitis.

**Case presentation:**

A 31-year-old Indian man presented with altered mental status, slight fever, and ataxia. Brain magnetic resonance imaging noted a widespread involvement of the white matter associated with slight cortical and subcortical damage in absence of contrast enhancement. An extensive infectious screening resulted negative while autoimmune analysis revealed the presence of MOG-Abs, detected with live cell-based assay. After treatment with intravenous immunoglobulins a marked and prompt clinical and radiological improvement was observed.

**Conclusions:**

To date, several areas of uncertainty still remain regarding clinical features and prognosis of subjects with MOG-Abs. The description of atypical cases is crucial, since recognition of this condition leads to prompt treatment and better prognosis, as in the case here reported.

## Background

Anti-myelin oligodendrocyte glycoprotein antibodies (MOG-Abs) have been reported in different inflammatory demyelinating diseases as acute disseminated encephalomyelitis (ADEM), neuromyelitis optica spectrum disorders (NMOSD), idiopathic optic neuritis, idiopathic myelitis, and atypical multiple sclerosis [1–13]. However, the whole spectrum of clinical phenotypes associated with MOG-Abs-related disorders has still to be clearly defined. Here, we present a patient with MOG-Abs and a clinical picture resembling infectious encephalitis as a possible new clinical phenotype associated with these antibodies.

## Case presentation

**Fig. 1:**
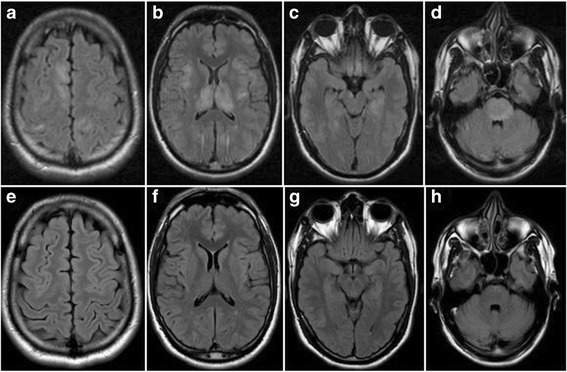
FLAIR sequence from the brain MRI at onset (**a**-**d**) and after treatment (**e**-**h**). Cortical and subcortical damage (**a**-**b**) with severe bilateral involvement of thalamus and internal capsule (**b**), mesial temporal lobes (**c**) and pons (**d**) in absence of contrast enhancement, is seen on brain MRI performed ad onset. After treatment with IVIg a significant improvement was noted with an almost complete resolution of the cortical, thalamic and basal ganglia involvement (**e**-**f**) and a reduction of temporal (**g**) and brainstem lesions (**h**)

**Fig. 2:**
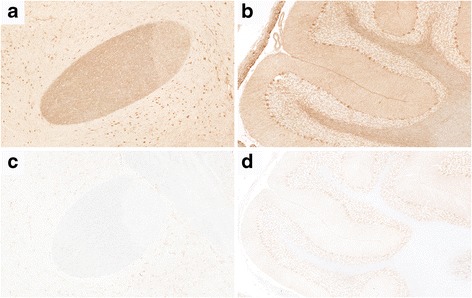
Immunohistochemical analysis. Serum (dilution 1:100, MOG-IgG and IgG1 titer 1:5120, also reactive with rat and mouse MOG) and CSF (dilution 1:2, MOG-ab titer 1:8) samples were screened by immunohistochemistry on a tissue-based assay (in-house; avidin-biotin-peroxidase technique; rat brain) as described previously by Sepulveda et al. 2016 [15]. The CSF (diluted 1:2) showed a specific myelin staining in the anterior commissure (**a**) and cerebellar white matter (**b**), whereas a control sample was negative (**c**, **d**). Magnification: A, C: ×100; B, D: ×60

A previously healthy 31-year-old Indian man presented to the emergency room with confusion and altered consciousness. Five days prior to onset of neurological symptoms he developed slight fever (<38 °C), sore throat and headache. His past medical history was unremarkable except for a recent stay in India for 2 months. Neurological examination disclosed a wide-based and unsteady gait and reduced level of consciousness. Diffuse papules and enanthema were also observed and spontaneously disappeared few days later. A slight increase of erythrocyte and leukocyte cell counts (13.000 cells/μL), erythrocyte sedimentation rate (30 mm/h) and C-reactive protein (48 mg/l) was noted on basic laboratory test. The patient was initially treated with acyclovir and ceftriaxone for a presumptive diagnosis of infectious encephalitis. A cerebrospinal fluid (CSF) analysis showed total protein level of 53 mg/dl, 179 cells/μL (90% mononuclear) in absence of oligoclonal IgG bands. On brain magnetic resonance imaging (MRI), fluid attenuated inversion recovery (FLAIR) and T2 diffuse hyperintensities involving thalamus, basal ganglia, internal capsule, mesial temporal lobes and brainstem associated with small hyperintensities in the subcortical, periventricular and cortical regions were noted in absence of contrast enhancement (Fig. 1a-d). Diffusion weighted images showed a mild restrictive pattern of the sopratentorial lesions affecting the left and right thalamus, the posterior limb of the internal capsule of both sides, the splenium and the posterior profile of the left trigonum. These lesions also presented medium-high apparent diffusion coefficient values and central core of low apparent diffusion coefficient, suggesting an inflammatory process. Whole spinal cord MRI was normal. A comprehensive workup for viral encephalitis and atypical infections including polymerase chain reaction for *Human immunodeficiency virus, Enterovirus, Herpes simplex 1–2-6, Epstein-Barr, Cytomegalovirus, Varicella Zoster, Toxoplasma, Mycobacterium tuberculosis, Treponema pallidum, Bartonella henselae, Human Parechovirus, West Nile, Dengue, Chikungunya,* and *Japanese encephalitis* resulted negative and also cultures for bacteria and fungi. An extensive autoimmune screening including anti-nuclear antibodies, complement levels, thyroid function and antibodies, autoantibodies to synaptic receptors and neuronal cell surface proteins was also negative. The criteria for collagen diseases, vasculitis, Behçet and Hashimoto encephalopathy were not satisfied. No neuropil staining was observed on tissue-based screening with immunohistochemistry on rat sections. Testing for MOG-Abs with a live cell-based assay with recombinant full-length MOG expressed in HEK293 cells [14], identified MOG-Abs both in the serum (titer of 1:5120) and in the CSF (titer 1:8). Staining of rat brain tissue with serum and CSF samples resulted in a specific myelin staining already described for MOG-Abs as shown in Fig. 2 [15]. The patient was treated with intravenous immunoglobulin (IVIg) 0.5 g/kg/day for 5 days with an almost complete clinical recovery in a few days. On control MRI examination after 2 weeks a dramatic improvement of pre-existing lesion was observed (Fig. 1e-h). Only the persistence of slight unsteady gait was noted at the last clinical evaluation 2 weeks after the onset.

## Discussion and conclusions

We here report an atypical presentation of MOG-Abs associated disorder with a prompt response to IVIg which satisfied the criteria of “possible encephalitis of presumed infectious origin” which also was the main presumptive clinical diagnosis [16]. Since the patient presented with subacute onset of altered mental status, new focal central nervous system findings and CSF pleocytosis in absence of alternative causes, he also met the revised diagnostic criteria for possible autoimmune encephalitis recently proposed [17]. However, the absence of neuropil staining on immunohistochemistry ruled out the presence of other well defined auto-antibodies or overlapping syndromes. In the case here reported, some features, as the multiple sopratentorial lesions of the white matter, basal ganglia, thalamus and brainstem, resemble those observed in ADEM. However, pleocytosis was higher than that usually observed in patients with ADEM and MRI was not totally compatible due to the type of gray and periventricular involvement and the absence of spinal cord lesions. Since criteria for ADEM have been clearly established only for pediatric cases [18] and the diagnosis in adults, also with MOG-Abs, remain challenging [17], we classified the case as “encephalitis” rather than ADEM.

Only five cases of encephalitis with MOG-Abs have been reported so far and are summarized in Table 1 [19, 20].

**Table 1:** Clinical, radiological and CSF data of the index case (case 1) compared with previously cases reported by Fujimori et al. (case 2) and Ogawa et al. (cases 3–6) with MOG-Abs and a presumptive diagnosis of encephalitis [19, 20]

Case	1	2	3	4	5	6
Onset	fever, headache, confusion, altered consciousness, unsteady gait	dizziness	eye pain and visual loss	seizure	involuntary movemet	headache and abnormal behavior
Seizures	no	yes	yes	yes	yes	yes
Optic neuritis	no	yes	yes	yes	no	no
Myelitis	no	no	no	no	no	no
CSF	increased cells and proteins	increased cells, negative at a second control	increased cells	increased cells	increased cells and proteins	increased cells and proteins
First Brain MRI	subcortical, periventricular, cortical, white matter, thalamus, basal ganglia, internal capsule, pons	frontal cortex	frontoparietal cortex	frontoparietal cortex	parietal cortex	emisphere cortex
Second Brain MRI	reduction of preexisting lesios	corpus callosum, cingulate gyri, frontal lobes	negative	optic nerve, emispheric cortex	negative	negative
Third Brain MRI		third ventricle, cerebral aqueduct, thalamus, nucleus basalis		negative		
Fourth Brain MRI		reduction of preexisting lesios				
Clinical symptoms	wide-based unsteady gate, reduced level of consciousness, diffuse skin swelling and pustules with enanthema	headache, paraparesis/paraplegia, memory decline, lethargy	delirium, paranoia, hallucination, anorexia	eye pain, visual loss, dysuria	headache, disorientation	agitation, violent behavior, delirium, emotional incontinence, aphasia, hemiparesis
Treatment	acyclovir, antibiotics, immunoglobulins	methylprednisolone and acyclovir, oral prednisolone	methylprednisolone, prednisolone, antiepilepsy drugs	methylprednisolone, prednisolone, antiepilepsy drugs	acyclovir, antibiotics, antimycotic, dexamethasone, prednisolone, antiepilepsy drugs	methylprednisolone, prednisolone, antiepilepsy drugs
Relapses	no	yes	yes	no	no	yes
Outcome	almost full recovery	full recovery	full recovery	full recovery	full recovery	full recovery

In these reports an autoimmune aetiology was the main presumptive diagnosis while in the case here described clinical and radiological data were suggestive of an infectious process. Moreover, all the previously reported cases presented seizures at onset or during the course of the disease and 3 out of 5 patients had optic neuritis, a feature clearly associated with MOG-Abs. The radiological features here described are also unique and in part different from the ADEM-like [19] or unilateral cortical lesions [20] previously reported. In these cases the onset was characterised by an exclusive cortical damage and in only one patient the involvement of corpus callosum, cingulate gyrus, frontal lobes, midbrain, thalamus, and nucleus basalis was noted during the follow-up [19]. In our patient we observed at onset an extensive involvement of the thalamus, basal ganglia, internal capsule, mesial temporal lobes and brainstem associated with subcortical, periventricular and cortical damage. Finally, the prompt response to IVIg is also a peculiar finding, since the other cases had a complete recovery after steroids treatment that led to the definition of “steroid- responsive encephalitis “[20]. The role of infectious agent in triggering MOG-Abs production is still a matter of debate. However, the flu-like symptoms here reported are not unexpected since attacks are preceded by infections at least once in about 40% of MOG-Abs positive cases [1]. The infection could also be responsible for the blood-brain barrier breakdown that allows the entry of autoantibodies in the central nervous system. This report confirms that MOG-Abs might denote a disease entity in its own right and that the spectrum of MOG-Abs associated diseases is wider than NMOSD and ADEM. Since a prompt and adequate treatment can lead to a favorable outcome, clinicians should be aware of this condition, also in clinical pictures suggestive for infectious encephalitis.

## Abbreviations

ADEM: Acute disseminated encephalomyelitis
CSF: Cerebrospinal fluid
FLAIR: Fluid attenuated inversion recovery
IVIg: Immunoglobulin
MOG-Abs: Anti-myelin oligodendrocyte glycoprotein antibodies
MRI: Magnetic resonance imaging
NMOSD: Neuromyelitis optica spectrum disorders
